# Influence of the Deformation Degree on the Evolution of the Microstructure and Properties of Al-10.0Zn-2.7Mg-2.3Cu Alloy During Short-Flow Thermo-Mechanical Treatment

**DOI:** 10.3390/ma18030554

**Published:** 2025-01-26

**Authors:** Hao Li, Yongxing Zhao, Yuanchun Huang, Yu Liu, Junhua Cheng

**Affiliations:** 1 College of Mechanical and Electrical Engineering, Central South University, Changsha 410083, China; haolii@csu.edu.cn; 2 School of Metallurgy and Environment, Central South University, Changsha 410083, China; zhaoyxeasy@163.com; 3 Hunan InnoChina Advanced Materials Co., Ltd., Yueyang 414021, China; 4 Light Alloy Research Institute, Central South University, Changsha 410083, China; 223801020@csu.edu.cn; 5 State Key Laboratory of High Performance Complex Manufacturing, Central South University, Changsha 410083, China

**Keywords:** Al-Zn-Mg-Cu alloy, ultra-high strength, deformation degree, thermo-mechanical treatment, particle-stimulated nucleation, grain refinement, mechanical property

## Abstract

A simple short-flow thermo-mechanical treatment (TMT) named L-ITMT (consisting of three steps: solution, warm deformation, and solution) was applied to ultra-high-strength Al-10.0Zn-2.7Mg-2.3Cu alloy to study the influence of the deformation degree on the particle distribution, resolubility, microstructure evolution, recrystallization mechanism, formation and development of deformation bonds, and mechanical properties. Increasing the rolling deformation during the L-ITMT process can effectively break up the second phase at the grain boundary and promote its dissolution, which is beneficial to aging precipitation strengthening and improves the strength of the alloy. The dominant mechanism changes from recovery to recrystallization when the deformation degree reaches 80%. As the strain increases, the deformation band becomes flatter and eventually becomes nearly parallel to the RD direction, promoting the occurrence of geometric recrystallization or continuous recrystallization (CRX). Under high-strain conditions, the formation mechanisms of recrystallized grains include discontinuous recrystallization (DRX), CRX, and particle-stimulated nucleation (PSN), but the main contributions to the formation of large-area fine-grained bands are CRX and PSN. The results showed that as the deformation degree increased from 10% to 80%, the improvement of solid solubility and grain refinement in the short-flow TMT process increased the ultimate tensile strength (701 MPa), yield strength (658 MPa), and elongation (11.3%) of the alloy by 15.7%, 10.8%, and 842%, respectively. This shows that the short L-ITMT process has a synergistic effect in significantly improving the plasticity and maintaining the strength of this ultra-high strength Al-Zn-Mg-Cu alloy.

## 1. Introduction

Al-Zn-Mg-Cu series alloys are widely used in the military and aviation fields due to their low density and excellent mechanical properties [1,2,3]. With the rapid development of these fields, there is a demand for the mechanical properties of alloys to meet higher standards. On the one hand, researchers began to focus on high-composition and rare-earth-added Al-Zn-Mg-Cu alloys that can provide higher strength [4,5,6] and better formability [7]. On the other hand, researchers have focused on improving the overall performance of alloys through grain refinement [8,9,10]. The methods of grain refinement are equal-channel angular pressing (ECAP) [10,11,12], severe plastic deformation (SPD) [13,14,15], cryorolling [16,17], and especially thermo-mechanical treatment (TMT) [18,19,20]. Among them, TMT is an effective process for large-scale production.

It has been found that TMT has the effect of affecting the particle size and distribution and thus the recrystallization mechanism during deformation, thereby achieving the effect of refining the grains to improve the ductility of the 7xxx series commercial aluminum alloys [18,19]. The application of ITMT (intermediate TMT) to Al-Zn-Mg-Cu alloys can be traced back to the ISML-ITMT (ITMT designed by Istituto Sperimentale dei Metalli Leggeri of Italy) process developed by Ri-Russo et al. [21] in 1974. The main idea of the process is to obtain coarse MgZn_2_ particles through low-temperature homogenization, then accumulate alloy energy storage through warm deformation; finally, static recrystallization occurs during the solid solution process. The advantage of this process is that the Cr dispersoids precipitated during the recrystallization process hinder the continued growth of recrystallized grains. However, the difficulty of obtaining initial sheets of dissolved Cr and the drawbacks of deformation have hindered the widespread application of this process. Later, Waldman et al. [22] replaced low-temperature homogenization with multi-stage homogenization to form FA-ITMT (ITMT designed by Frankford Arsenal). Under such process conditions, the dissolution/precipitation of the element Cr no longer has a significant impact on the recrystallization of the alloy, but it results in a new problem of excessively long homogenization time. Based on previous research, Wert et al. [23] developed RI-ITMT, widely used in 7xxx commercial aluminum alloys, by introducing coarse MgZn_2_ particles through solid solution and over-aging. Paton et al. successfully applied the RI-ITMT (ITMT designed by Rockwell International Science Center) process to 7075 aluminum alloy and improved its ductility while increasing its corrosion resistance. Malek et al. [24] obtained fine grains with a size below 13 μm through the RI-ITMT process. Kumar et al. [25] applied RI-ITMT technology to 7010 alloy and obtained fine recrystallized grains with a size of about 10 μm. At the same time, the alloy exhibits superplasticity at high temperatures. In recent years, with a deeper understanding, TMT processes have been developed with shorter processes, less time consumption, and lower energy consumption. For example, Huo et al. [19] used warm rolling and continuous rolling instead of the over-aging and warm rolling in RI-ITMT to reduce the average grain size of sheets of 7075 aluminum alloy from 25 μm to 10 μm, which significantly improved the plasticity of the alloy.

The TMT process has been widely used in 7xxx-series commercial aluminum alloys, but there are few reports on high-content or ultra-high-strength Al-Zn-Mg-Cu alloys. Therefore, this article proposes a short-flow TMT process (L-ITMT, ITMT designed by Li) suitable for high-content aluminum alloys (including three processes: solid solution, warm deformation, and solid solution), and focuses on the evolution of the recrystallization mechanism during the process of increasing deformation and its influence on the mechanical properties of the alloy.

## 2. Experimental Details

**Figure 1:**
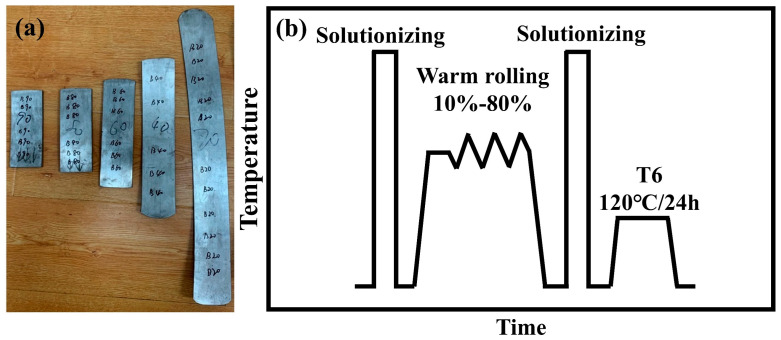
(**a**) The state of the plates after rolling and (**b**) a schematic diagram of the short-flow TMT process.

The materials used in this study were homogenized at 420 °C for 10 h and 470 °C for 24 h immediately after semi-continuous casting, and their chemical composition is shown in Table 1. The plates used in the test were taken from the middle of the homogenized bar and had dimensions of 10 mm × 50 mm × 70 mm. Figure 1b shows a schematic diagram of the short-flow TMT process. All plates were quenched immediately after solutionizing at 475 °C for 2 h. Afterwards, the plates were rolled with a deformation degree of up to 80% at 300 °C (as shown in Figure 1a). The specific process of rolling was as follows: First, the sample was kept in a furnace at 300 °C for 30 min; next, segmented rolling was carried out with deformation amounts of 10%, 10%, 20%, 20%, and 20%. The sample was returned to the furnace for insulation for 5 min between each rolling pass. All plates were solutionized (475 °C/2 h) and peak-aged (T6: 120 °C/24 h) after the final rolling process. The insulation during the rolling process and solid solution were carried out using aluminum alloy heat treatment furnace (Muffle furnace RX-650-25, Changsha, China). Its rated power, maximum heating temperature, and effective heating chamber size were 25 KW, 650 °C, and 400 mm * 300 mm * 300 mm, respectively. The diameters of the upper and lower rollers of the rolling mill were the same, at Φ100 mm. The maximum rolling pressure was 200 kN, and the roll speed was constant at 20 r/min. The applicable temperature range for the aging furnace (WGL-65D) was 10 °C to 300 °C, with a rated power of 1.6 kW and a temperature fluctuation of ±1 °C.

The microstructure of the material was characterized by scanning electron microscopy (SEM, TESCAN MIRA3, Brno, Czech Republic), and the proportion of related second phases is analyzed by Image Pro Plus software (6.0 version). The samples for SEM observation were polished step by step with water sandpaper, metallographic sandpaper, and diamond with a particle size of 1.0 μm until the surface of the sample is shiny and scratch-free. Then, the samples were observed on a TESCAN MIRA3 equipped with an energy-dispersive spectrometer (EDS, Oxford, UK). The size distribution and characteristics of grains and subgrains were analyzed by electron backscatter diffraction (EBSD), with step sizes of 0.2–4 μm. EBSD samples were prepared by electropolishing with a solution of 10% C_2_H_5_OH + 90% HClO_4_ at −20 °C for 20–30 s at 20 V after mechanical polishing. The EBSD results were analyzed using channel 5 software (2022 version). The testing of mechanical properties was carried out on a CMT5105 test machine (Shenzhen, China) at room temperature at a speed of 2 mm/min. Tensile specimens (40 mm × 6 mm) were cut along the RD direction from the sheet in the T6 state. Each sample must undergo at least three tensile tests to ensure reproducibility. The relevant data was plotted using Origin 2022 software.

## 3. Results

### 3.1. Second-Phase Distribution After Rolling

**Figure 2:**
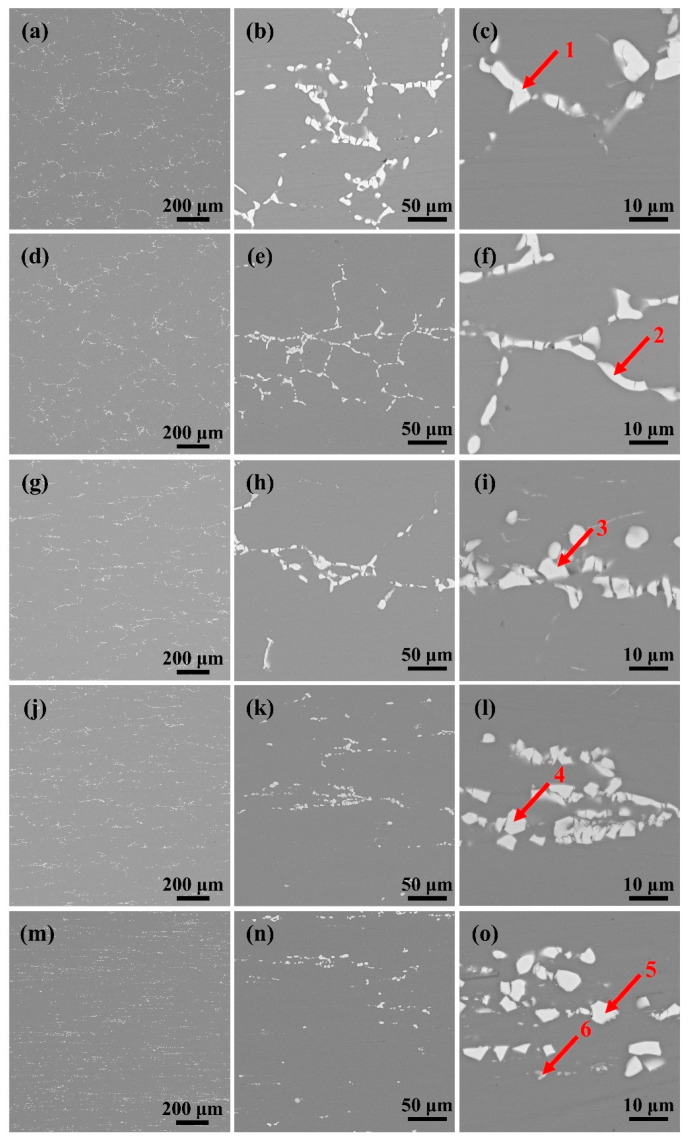
Distribution of the second phase of plates with different percentages of reduction after rolling: (**a**–**c**) 10%; (**d**–**f**) 20%; (**g**–**i**) 40%; (**j**–**l**) 60%; (**m**–**o**) 80%.

Figure 2 shows the SEM images of the second-phase distribution of the rolled sheets. The results of EDS showed that the main second phase was mainly T phase with very little Al_7_Cu_2_Fe (as shown in Figure 2 and Table 2). From a statistical perspective, the content of Al_7_Cu_2_Fe relative to the content of T phase can be neglected. When the deformation of the plate was less than 20%, the second phase was distributed in a network structure (Figure 2a,b,d,e), and the second phase between the grains began to break gradually (Figure 2c,f). When the reduction amount continued to increase to 60%, the reticular second phase gradually transformed into a fishbone distribution (Figure 2g,h,j,k), and the degree of fragmentation of the second phase between grains intensified, and it tended to gradually separate into single second phase particles (Figure 2i,l). When the reduction increases to 80%, the second phase of the fishbone distribution disappeared, replaced by a meteor distribution along the RD direction (Figure 2m,n), and the second-phase particles were diffusely distributed (Figure 2o).

### 3.2. Second-Phase Distribution After Solution

**Figure 3:**
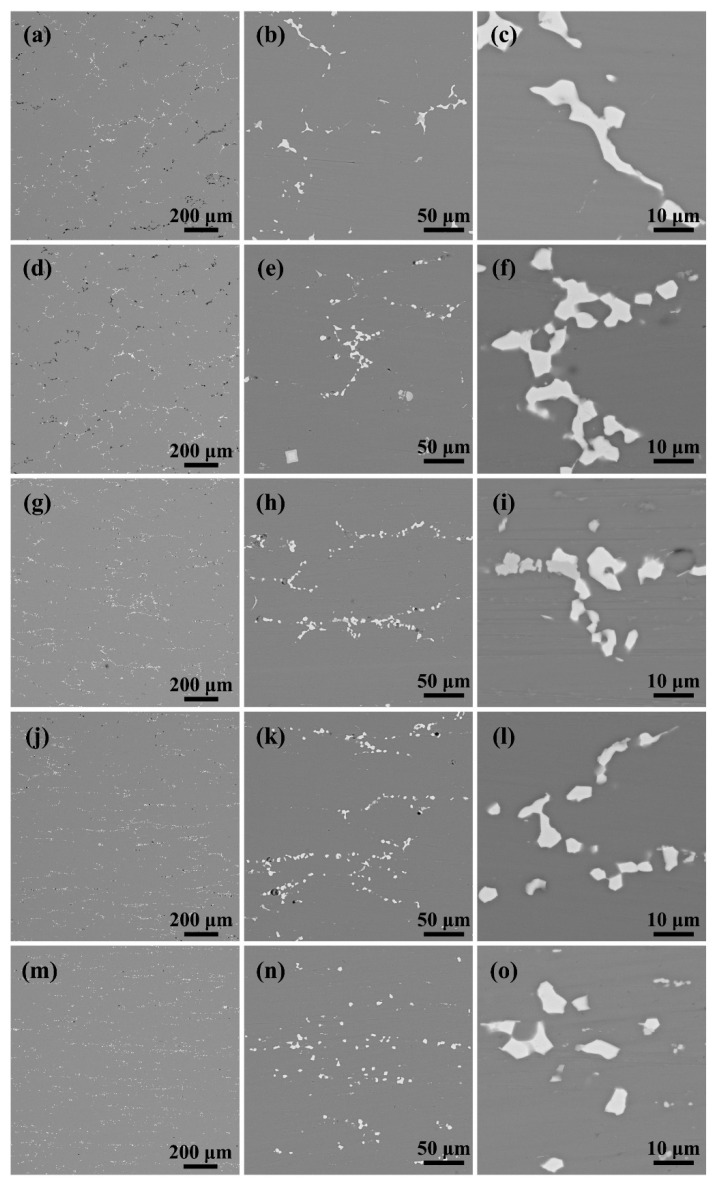
Distribution of the second phase of plates with different percentages of reduction after solution: (**a**–**c**) 10%; (**d**–**f**) 20%; (**g**–**i**) 40%; (**j**–**l**) 60%; (**m**–**o**) 80%.

After solution treatment at 475 °C/2 h for plates with different reduction, their overall distribution were similar to those after rolling, but the volume fraction was greatly reduced (Figure 2). As the amount of reduction increased, the remaining second phase increased slightly (from 1.09% to 1.40%), and the degree of overburning decreased significantly (from 1.20% to 0.07%, Figure 3a,d,g,j,m and Table 3). The above results indicate a general decreasing trend of the non-dissolved second phase with the increase in the reduction amount. As the rolling reduction increased, the second phase was broken into finer and more dispersed particles. Scholars [19,26] have explained the phenomenon that the finer phase has a higher degree of resolubility under the same solid solution conditions from the perspective of solute atom diffusion.

### 3.3. Recrystallization Differences After Solution

**Figure 4:**
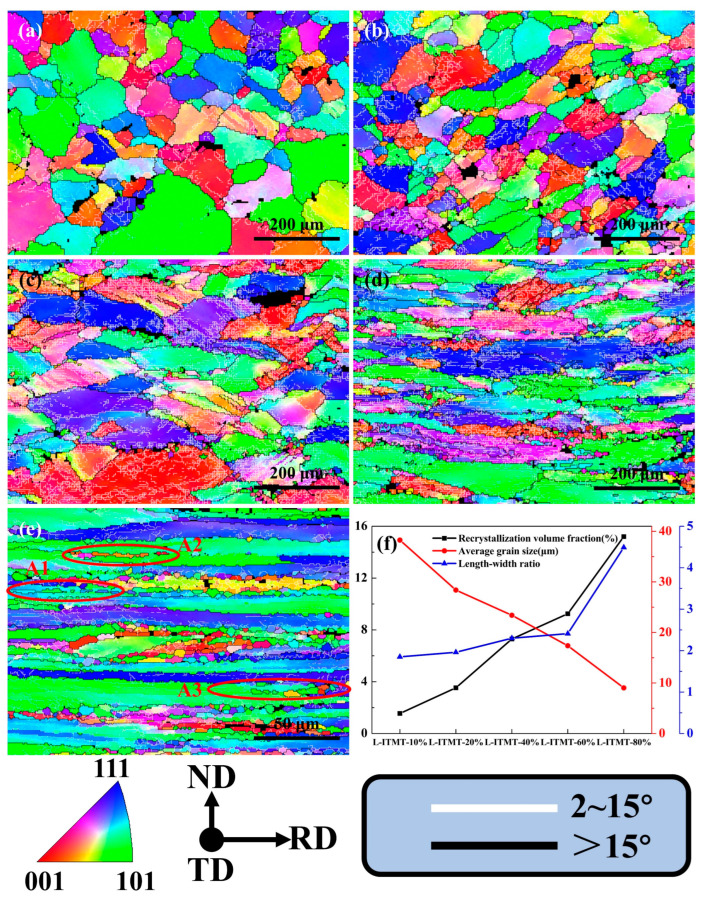
EBSD-IPF maps after solid solution with different percentages of rolling reduction: (**a**) 10%; (**b**) 20%; (**c**) 40%; (**d**) 60%; (**e**) 80%. (**f**) Statistics of recrystallized volume fraction, average grain size, and aspect ratio.

The recrystallization state of plates with different reductions after solid solution can be preliminarily analyzed from Figure 4. With the intensification of deformation, the morphology of the grains changed obviously. Under the rolling force, the grains were gradually flattened and elongated, forming a fibrous structure distributed along the rolling direction. In the process of increasing the reduction from 10% to 80%, the aspect ratio of the overall grain increased from 1.85 to 4.49 (Figure 4f and Table 4). The degree of recrystallization of the alloy was significantly improved (from 1.55% to 13.90%), which was mainly due to the continuous increase in the alloy’s storage energy during deformation, making it easier to recrystallize and promote the formation of fine grains during the solid solution process. The average grain size of the alloy also becomes smaller (from 38.27 μm to 9.04 μm). Additionally, many researchers [27,28,29] have conducted detailed studies on Al-Zn-Mg Cu alloys containing Zr and Sc, and the results have shown that the formed Al_3_ (Zr, Sc) precipitates can not only improve the strength of the alloy but also hinder the further growth of fine grains.

**Figure 5:**
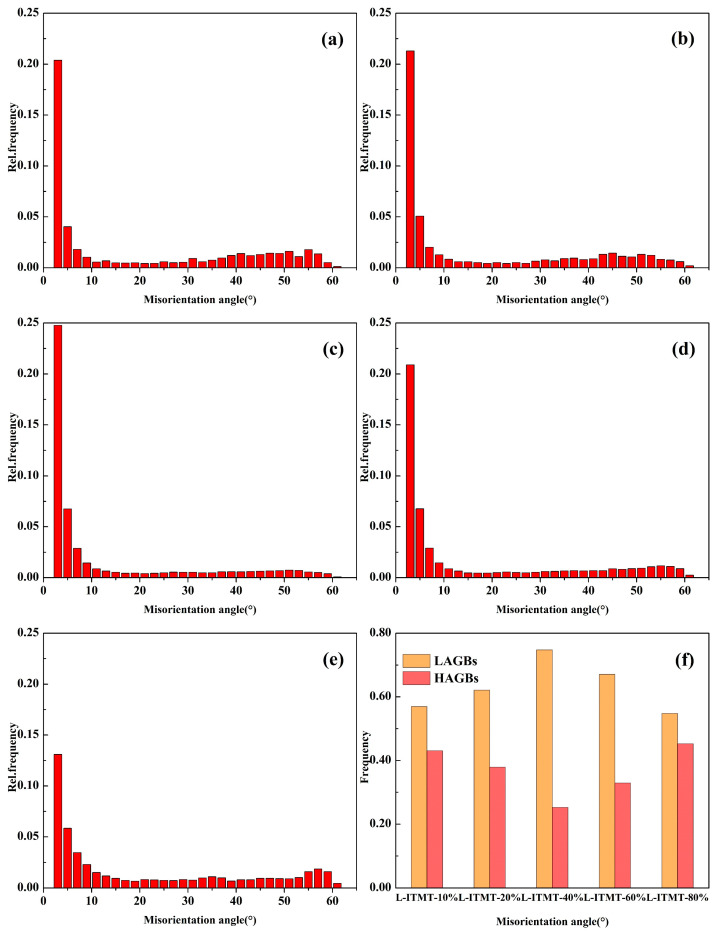
Misorientation distribution of different percentages of rolling reduction after solid solution: (**a**) 10%; (**b**) 20%; (**c**) 40%; (**d**) 60%; (**e**) 80%. (**f**) Statistics of fraction of low (high)-angle grain boundaries.

Through the statistics of the fraction of low-angle grain boundaries (LAGBs, Figure 5), it was found that with the increase in deformation, the fraction of LAGBs first increased and then decreased (Figure 5f). This is mainly attributable to the fact that the internal residual dislocations of the grains were retained more with the increase in strain, which led to a continuous increase in the total quantity of LAGBs. However, when the degree of deformation reached 40%, the LAGBs had covered almost all the grains (Figure 4c), and the total amount increased slightly in the subsequent deformation process from a statistical point of view, while the proportion of high-angle grain boundaries (HAGBs) of the fine grains by recrystallization was increasing. As a result, the proportion of LAGBs evolved as described above.

**Figure 6:**
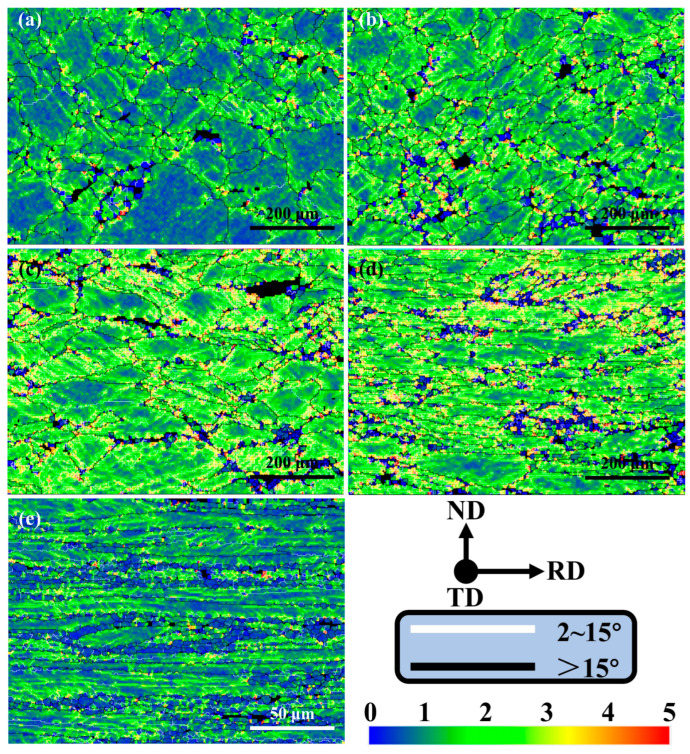
KAM maps after solid solution with different percentages of rolling reduction: (**a**) 10%; (**b**) 20%; (**c**) 40%; (**d**) 60%; (**e**) 80%.

The kernel average misorientation (KAM) diagram after solid solution with different rolling reductions can intuitively reflect the distribution of residual stress and stored energy, as shown in Figure 6. The color bar in the KAM diagram changes from blue to green and then to red, indicating that the local misorientation increases from 0° to 5°. In order to observe the state of local misorientation near HAGBs and LAGBs more conveniently, they are marked with black and white lines in the KAM maps, respectively. It can be seen from Figure 6 that, regardless of the degree of deformation, the interior of the fine recrystallized grains is dark blue, indicating that the internal energy storage of the small grains is close to zero. The recrystallization process in the solid solution process consumes the dislocations around it, which reduces the local misorientation. With the increase of the degree of deformation, the number of fine, dark blue grains increases, which also indicates the increase of the degree of recrystallization.

Generally, the interior of the large grains is light blue, while the grain boundaries appear green, yellow or even red, which indicates that dislocations are more likely to accumulate near the HAGBs during the previous rolling process. In addition, the colors near the deformation zone inside the grains are mainly yellow and green, and gradually transition from green to yellow and red with the increase of rolling reduction, which indicates that a higher degree of deformation is more conducive to the accumulation of dislocations near the deformation zone.

**Figure 7:**
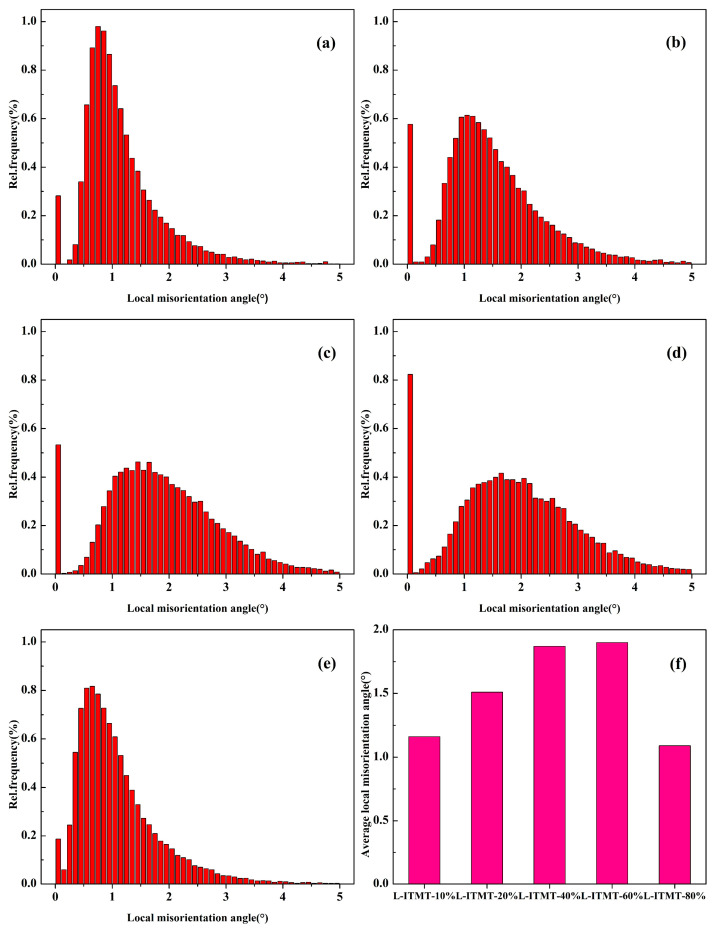
Local misorientation distribution after solid solution with different percentages of rolling reduction: (**a**) 10%; (**b**) 20%; (**c**) 40%; (**d**) 60%; (**e**) 80%. (**f**) Average local misorientation.

As the rolling reduction increases from 10% to 60%, the proportions of green, yellow, and red increase (Figure 6a–d). It can be observed that the peaks in the local misorientation diagram are gradually shifting to higher angles (Figure 7a–d), and the statistical results show that the average value increases from 1.16 to 1.90 (Figure 7f). When the reduction continues to increase to 80%, the KAM map generally returns to the blue-green distribution (Figure 6e), and the statistical results also show that the average local misorientation decreases to 1.09 (Figure 7e,f). This indicates that the dislocations accumulated in the rolling process undergo a more complete recrystallization during the solid solution process, and transform into more fine recrystallized grains with low dislocation density. Therefore, it can be considered that when the deformation reduction is less than 60%, the alloy is mainly based on the recovery mechanism. When the deformation reduction exceeds 80%, recrystallization is the main mechanism.

### 3.4. Mechanical Properties After Thermo-Mechanical Treatment

**Figure 8:**
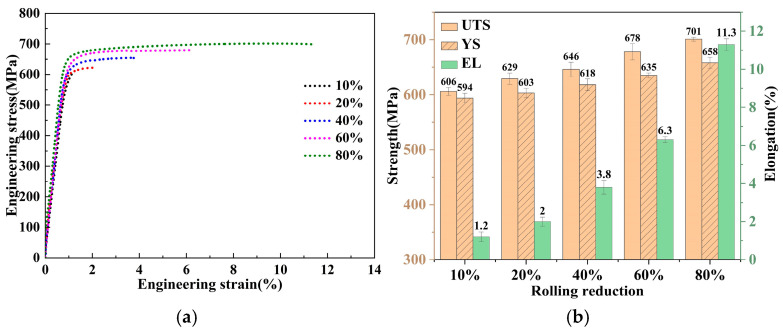
(**a**) Stress–strain curve and (**b**) mechanical properties after L-ITMT.

Figure 8a,b shows the stress–strain curves and corresponding mechanical properties of the final state under different degrees of deformation in the L-ITMT process. During the process of increasing the deformation degree from 10% to 20%, the ultimate tensile strength (UTS) of the board increased from 606 MPa to 629 MPa, the yield strength (YS) increased from 594 MPa to 603 MPa, and the elongation (EL) increased significantly from 1.2% to 2.0%. Under moderate deformation levels of 40% and 60%, the comprehensive mechanical properties of the board were further improved. Their UTS values were 646 MPa and 678 MPa, their YS values were 618 MPa and 635 MPa, and their EL values were 3.8% and 6.3%, respectively. When the recrystallization mechanism underwent a transformation (as described in Section 3.3), that is, when the degree of deformation reached 80%, the UTS and YS of the alloy increased to 701 MPa and 658 MPa, respectively, while the EL increased significantly to 11.3%.

In a word, as the deformation degree increased from 10% to 80%, the short-flow TMT process increased the UTS, YS, and EL of the alloy by 15.7%, 10.8%, and 842%, respectively.

## 4. Discussion

### 4.1. The Formation and Development of the Deformation Bonds

**Figure 9:**
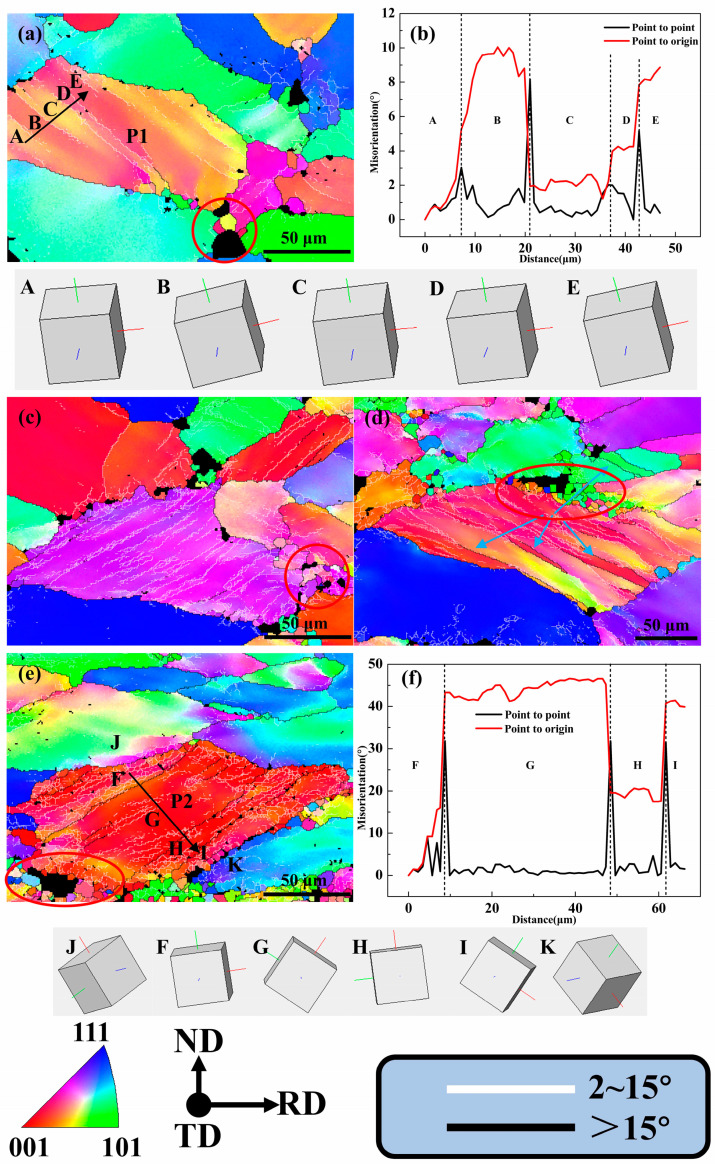
Enlarged view of local area in Figure 5 and misorientation variation along the direction of the arrow: (**a**) local area in Figure 5a; (**b**) misorientation variation of (**a**); (**c**) local area in Figure 5b; (**d**) local area in Figure 5c; (**e**) local area in Figure 5d; (**f**) misorientation variation of (**e**).

Due to the inhomogeneous stress transmitted by adjacent grains or the inherent instability of grains during plastic deformation, individual grains in the alloy are massively subdivided into domains with different orientations during deformation. The deformed bands are partially retained during the solid solution process. As shown in Figure 4a, when the deformation was only 10%, obvious deformation bands composed of parallel LAGBs had been formed inside a few grains. An enlarged view of one of the grains G1 containing deformation bands is shown in Figure 9a. By analyzing the misorientation from point to point and from point to origin from region A to region E, it can be concluded that the misorientation of different deformation zone regions was less than 10° (Figure 9b). In addition, the orientation of each region can be intuitively observed through the Euler angles of regions A–E. The orientations were very similar between regions A and C and between regions B and E, which indicates that the applied strain was accommodated by a set of slip systems, and each region rotated at a small angle.

When the degree of deformation increased to 20%, deformation bands composed of LAGBs could be observed in about half of the grains (Figure 4b). Furthermore, when the degree of deformation increased to more than 40%, the deformation band covers almost all the grains (Figure 4c–e), which shows that the increase in strain was very conducive to the formation of a deformation band. When the degree of deformation reached 40%, some deformation bands composed of HAGBs could be clearly observed inside the grains, as shown in the position marked by the blue arrow in Figure 9d. When the rolling reduction continued to increase to 60%, a HAGB deformation zone running through the grains could be observed, and the original grain P2 was divided into several new grains with higher aspect ratios (Figure 9e). This is also one of the important reasons for the decrease in the average grain size and the increase in the grain aspect ratio with the increase in strain.

The following two points can prove that the F-I regions were new grains formed by shearing and originally belonged to the grain P2. First, the analysis of the adjacent misorientation and cumulative misorientation of the region F to region I (Figure 9f) showed that the misorientation of the two adjacent regions was higher than 20°, and the misorientation of the F and G regions even reached 50°. They all belonged to HAGBs, which can be considered evidence of the formation of new grains. Second, although the F, G, H, and I regions inside P2 had large orientation differences, their orientation was <111>//TD. It can be considered that the internal deformation bands of P2 grain consisted of torsion to a large extent under the action of shearing. At the same time, the orientations of the J region adjacent to the F region and the K region adjacent to the I region were <123>//TD and <112>//TD, which are completely different from the orientations of the F~I regions. Therefore, it is concluded that the new grains F~I belonged to the original grain P2.

When the degree of deformation continued to increase to 80%, the deformation band also continued to elongate along with the grain, and the direction was almost parallel to the RD direction (Figure 4e). We will discuss how the elongated deformation bands affect the recrystallization to form fine grains in the subsequent subsections.

### 4.2. Geometric Recrystallization

When the degree of deformation gradually increases, the width of the deformed grains or bands in the ND direction gradually decreases to a size close to that of a single subgrain after rolling. Subsequent small grain boundary movements can result in fine grains surrounded by HAGBs. Such fine grains have no clearly identifiable “nucleation” and “growth” stages, and uniform evolution occurs in the local structure of the material, which can be classified as continuous recrystallization (CRX) or geometric recrystallization (GRX).

**Figure 10:**
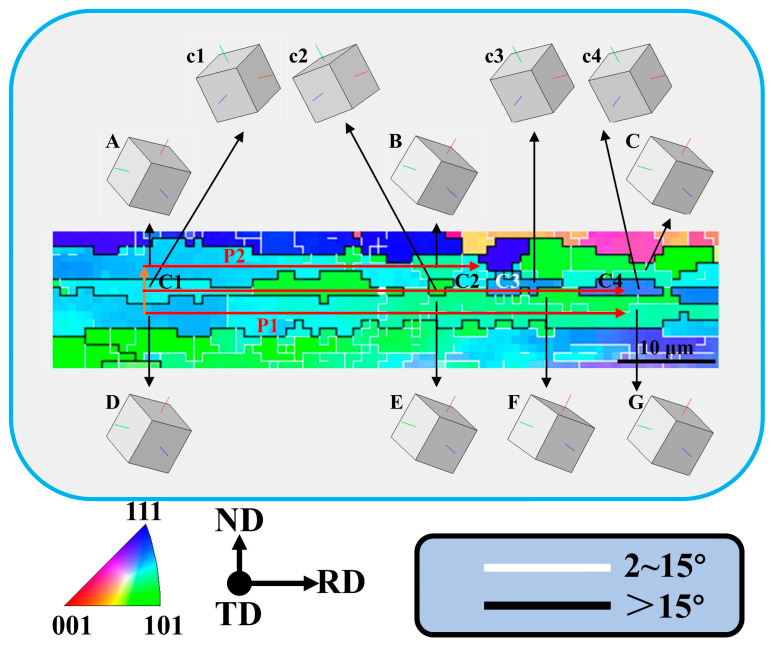
Local area in Figure 5e.

**Figure 11:**
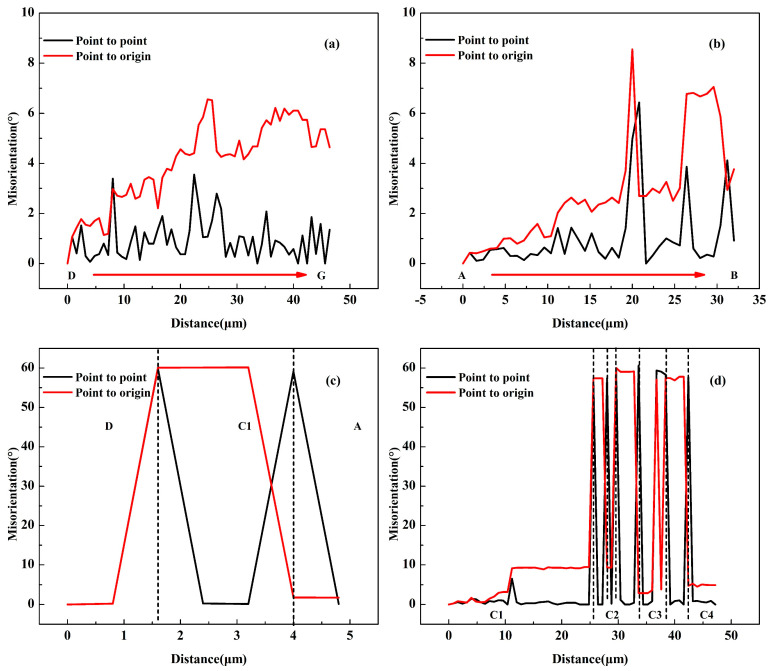
Misorientation along the arrow direction in Figure 2: (**a**) Area D to Area G; (**b**) Area A to Area B; (**c**) Area D to Area A; (**d**) Area C1 to Area C4.

As shown in Figure 4e,f and Table 4, when the rolling reduction reached 80%, the average aspect ratio of grains reached 4.49, which meets the conditions (aspect ratio greater than 4) for GRX [30]. This phenomenon was observed in many areas of its microstructure, as shown in the A1~A3 area in Figure 4e. As mentioned in Section 3.1, the deformed bands with HAGBs formed by shearing were almost distributed along the RD direction at a rolling reduction of 80%. The width along the ND direction was about 2 μm (Figure 10), which is very close to the grain size of a single subgrain. The orientations of the P2 region (position A~C) and the P1 region (position D~G) were very close in terms of cubic lattice (Figure 10), and neither the point-to-point difference nor the point-to-origin difference exceeded 9° (Figure 11a,b), which indicates that both the P1 and P2 regions belonged to an original grain (named grain P). The point-to-point misorientation and point-to-origin misorientation from the D area to the A area indicate that the misorientation of C1, P1, and P2 was 60° (Figure 11c), but the orientation of all three was <123>//TD. Therefore, C1 was originally part of the deformation band formed inside the grain P. Figure 11d shows that the misorientation between C1~C4 grains was less than 10°, and it can be more clearly observed that their orientations are very close from orientations c1~c4 represented by their cubic lattice. They originally belonged to the same deformation band during the deformation process, and the grain boundaries were twisted at a small angle during the solid solution process to form new grains with close orientations. Therefore, the fine C2~C4 grains distributed along the RD direction can be considered the final result of the development of the original elongated deformation band through GRX.

### 4.3. Discontinuous Recrystallization

**Figure 12:**
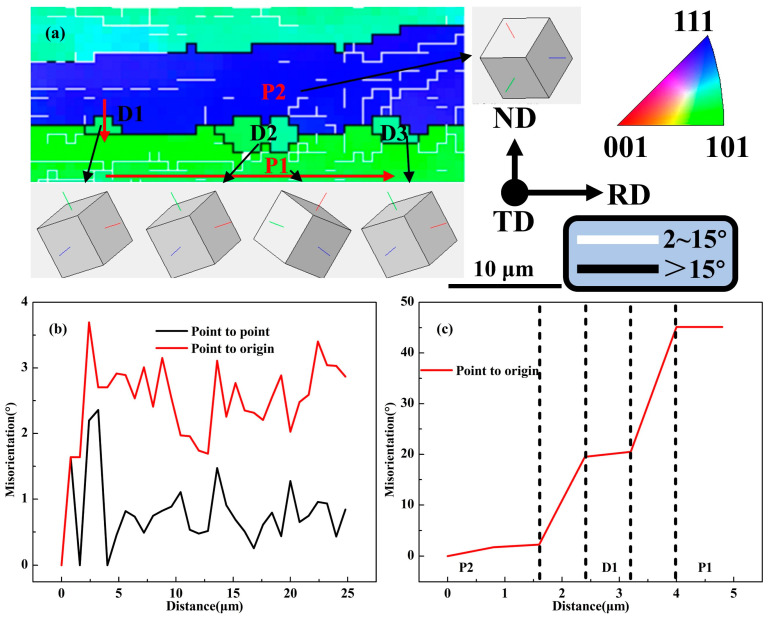
(**a**) Local area in Figure 5e, (**b**) misorientation in the direction of the red arrow inside the P1 grain, and (**c**) misorientation through D1 in the direction of the red arrow.

The variation of orientation misorientation along the RD direction of P1 grains is shown in Figure 12a,b. The point-to-point misorientation was less than 3°, and the point-to-point initial misorientation did not exceed 4°, which indicates that the residual dislocation energy storage of P1 grains after solid solution was very low. The orientations of the D1, D2, and D3 grains at the grain boundary represented by the cubic lattice were very close, and a large-angle rotation occurred compared with the P1 grain below. Due to their symmetry, the orientations of the recrystallized grains D1~D3 and the P1 grains were all similar to <123>//TD directions. This indicates the presence of another recrystallization mechanism, discontinuous recrystallization (DRX), during the solution process after 80% rolling reduction. This mechanism is different from the randomly oriented recrystallized grains induced by CRX, DRX usually occurs near HAGBs, and its typical feature is induced by irregular zigzag grain boundary migration. With the further development of the migration, the grain boundary bows out, and finally the DRX fine grains with no internal dislocation energy storage are formed. The point-to-origin misorientation changes along the ND direction passing through the D1 grains are shown in Figure 12c. The misorientations between P2 and D1, between D1 and P1, and between P2 and P1 grains were about 20°, 25°, and 45°. This shows that the misorientation formed a gradient along P1 to P2, which was the key cause of D1 grains bowing and nucleating from P1 to P2.

### 4.4. Particles Stimulate Nucleation

Several studies [19,23] have shown that large particles can act as preferential nucleation sites, a well-known phenomenon called particle-stimulated nucleation (PSN). In this study, it was observed that the gradual increase in deformation had an impact on the PSN, as shown in Figure 9. When the deformation degree was only 10%, a small number of recrystallized grains could be observed around large particles after solid solution, as shown in the position marked with a red circle in Figure 9a. This shows that even under low strain, dislocations could be effectively pinned around the particles and PSN occurs during the subsequent solid solution process, resulting in a small quantity of recrystallized grains. As the degree of deformation increased, the accumulated dislocation energy storage around the second phase increased, which increased the number of sites where PSN occurred (as shown in Figure 5a–e), and the number of recrystallized grains around coarse particles increased significantly, as shown in Figure 9a,c–e. This indicates that increased strain or increased energy storage can promote the generation of PSN.

### 4.5. Formation of Fine-Grained Bands

**Figure 13:**
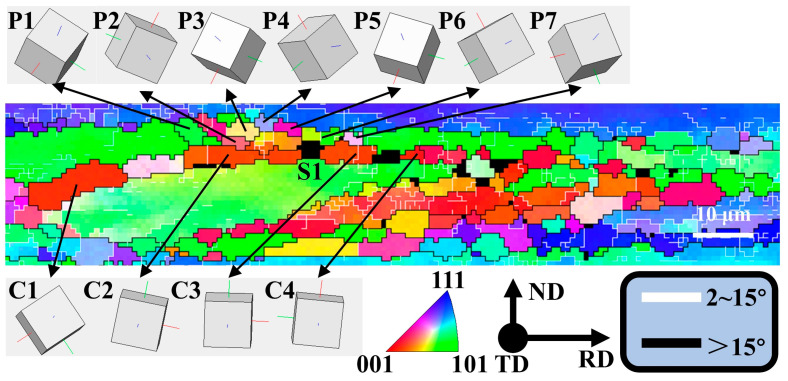
Enlarged view of the fine-grained band area in Figure 5e.

Under the condition of rolling reduction of 80%, obvious fine-grained bands were formed in the middle area of Figure 4e, while no obvious fine-grained bands were observed within 60% reduction. This indicates that the primary need for producing fine-grained bands is under high strain or high energy storage. Figure 13 shows a local enlargement of the fine-grained region in Figure 4e, where multiple coarse particles are found around the fine grain band. The cubic lattice orientation of the fine grains P1~P7 around the coarse particle S1 was quite different, which is due to the fact that the coarse particle S1 produced randomly oriented fine grains through the PSN effect during the solution process. This is one of the characteristics of formation of the fine-grained bands. On the other hand, the orientations of the C1~C4 grains distributed along the RD direction around particle S1 were not completely consistent, but their orientations were approximately <001>//TD direction. This indicates that they were formed through the twisting of deformation bands and belong to the continuous recrystallization mechanism. Coarse particles can also increase the formation rate of high angle grain boundaries by disrupting the flatness of grain boundaries and establish new high angle grain boundaries by rotating the lattice around the coarse particles. This reduces the width of the surrounding large grains or deformation bands extending in the ND direction, making narrow grains or deformation bands near the particles more prone to continuous recrystallization during the solid solution process.

**Figure 14:**
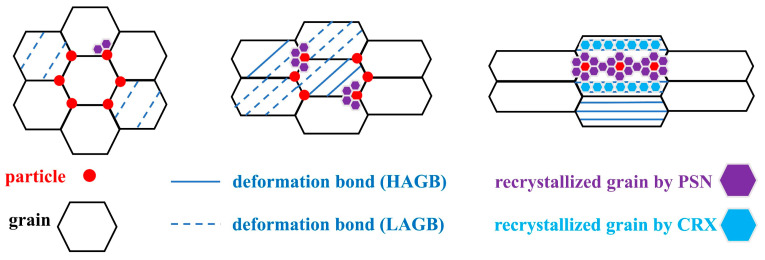
Formation mechanism of fine-grained bands.

In summary, fine grain bands can be considered to be caused by the PSN effect of multiple adjacent coarse particles and the fine grains distributed in the RD direction generated by the continuous recrystallization mechanism (as shown in Figure 14).

### 4.6. Effect of Microstructure on Mechanical Properties

As the deformation degree increased, the overburned second phase decreased and the dissolved second phase increased under the same solid solution conditions (as shown in Figure 4). Therefore, the matrix solid solubility was higher, which was beneficial to the subsequent aging precipitation strengthening.

In addition, as the deformation of the plate increased, the PSN effect became more significant. At the same time, the continuous recrystallized grains produced in the process of narrowing of the deformation bands increased. The combination of the two mechanism eventually formed a large-area fine-grained zone (as shown in Figure 14), resulting in a significant reduction in the average grain size (from 38.27 μm to 9.04 μm, statistics in Table 4). Many researchers [31,32,33] have demonstrated the benefits of grain refinement in improving ductility, as more uniform slip can occur and more dislocations can be stored in the fine grain structure, thereby promoting higher strain hardening ability to delay fracture and increase elongation.

## 5. Conclusions

(1) Increasing the rolling deformation from 10% to 80% during the short-flow thermo-mechanical treatment (TMT) process can effectively break up the second phase at the grain boundary, which effectively reduces the overburned second phase from 1.20% to 0.07% during the solid solution process. At the same time, the amount of dissolved second phase increases by 0.82%, which is very beneficial to the subsequent aging precipitation strengthening and improves the strength of the alloy.
(2) Based on a comprehensive analysis of the grain size, kernel average misorientation (KAM) diagram, and recrystallization volume fraction, it is concluded that when the deformation amount in the L-ITMT process reaches 60%, the dominant mechanism changes from recovery to recrystallization.
(3) During the process of increasing the deformation degree from 10% to 80%, the ultimate tensile strength (UTS) of the board increases from 606 MPa to 701 MPa, the yield strength (YS) increases from 594 MPa to 658 MPa, and the elongation (EL) increases significantly from 1.2% to 11.3%.
(4) As the deformation increases, the deformation bands gradually transform from LAGBs to HAGBs and become flat. When the rolling reduction reaches 80%, the average aspect ratio of grains reaches 4.49 (the deformation band is nearly parallel to the RD direction and has a higher aspect ratio), which promotes the conditions for geometric recrystallization (GRX) or continuous recrystallization (CRX).
(5) The particle-stimulated nucleation (PSN) effect can be observed at a deformation degree of 10% and becomes more significant with increasing strain. When the degree of deformation reaches 80%, the PSN effect is the main contributor to fine grains.
(6) Under high-strain conditions, the formation of recrystallized grains includes three mechanisms, namely, discontinuous recrystallization (DRX), CRX, and PSN, but the main contributors to the formation of large-area fine-grained bands are CRX and PSN. This effectively reduces the average grain size (from 38.27 μm to 9.04 μm) of the alloy, slightly increases the strength of the alloy, and greatly improves the plasticity of the alloy.

## Figures and Tables

**Table 1:** Chemical composition of Al-10.0Zn-2.7Mg-2.3Cu-0.11Zr-0.12Sc (wt%).

Element	Zn	Mg	Cu	Zr	Sc	Al
wt%	10.09	2.73	2.28	0.11	0.12	Bal

**Table 2:** Chemical composition of the phases in Figure 2 (at%).

Point	Al	Cu	Zn	Mg	Fe	Identified Phase
1	39.19	13.35	21.07	26.39	0.00	T
2	39.72	12.78	24.63	22.87	0.00	T
3	35.66	14.09	22.71	27.54	0.00	T
4	31.35	7.24	28.96	32.45	0.00	T
5	36.45	9.81	28.39	25.35	0.00	T
6	72.51	13.41	4.96	3.78	5.34	Al_7_Cu_2_Fe

**Table 3:** Statistics of the second-phase volume fraction after solid solution of plates with different percentages of reduction (%).

Reduction	Remaining Second Phase	Burnt Second Phase	Undissolved Second Phase
10%	1.09	1.20	2.29
20%	1.23	0.75	1.98
40%	1.34	0.49	1.83
60%	1.39	0.27	1.68
80%	1.40	0.07	1.47

**Table 4:** Statistics of recrystallization volume fraction (VF_Rx_), aspect ratio, fraction of low-angle grain boundaries (F_LAGBs_), fraction of high-angle grain boundaries (F_HAGBs_), and average grain size under different percentages of rolling reduction after solid solution.

Rolling Reduction	VF_Rx_ (%)	Aspect Ratio	F_LAGBs_ (%)	F_HAGBs_ (%)	Average Grain Size (μm)
10%	1.55	1.85	0.57	0.43	38.27
20%	3.53	1.96	0.62	0.38	28.38
40%	7.30	2.30	0.75	0.25	23.40
60%	9.25	2.41	0.67	0.33	17.37
80%	13.90	4.49	0.55	0.45	9.04

## Data Availability

The original contributions presented in this study are included in the article. Further inquiries can be directed to the corresponding authors.
